# Frequent Words Do Not Break Continuous Flash Suppression Differently from Infrequent or Nonexistent Words: Implications for Semantic Processing of Words in the Absence of Awareness

**DOI:** 10.1371/journal.pone.0104719

**Published:** 2014-08-12

**Authors:** Tom Heyman, Pieter Moors

**Affiliations:** Laboratory of Experimental Psychology, University of Leuven, Leuven, Belgium; Birkbeck College, United Kingdom

## Abstract

Continuous flash suppression (CFS) has been used as a paradigm to probe the extent to which word stimuli are processed in the absence of awareness. In the two experiments reported here, no evidence is obtained that word stimuli are processed up to the semantic level when suppressed through CFS. In Experiment 1, word stimuli did not break suppression faster than their pseudo-word variants nor was suppression time modulated by word frequency. Experiment 2 replicated these findings, but more critically showed that differential effects can be obtained with this paradigm using a simpler stimulus. In addition, pixel density of the stimuli did prove to be related to suppression time in both experiments, indicating that the paradigm is sensitive to differences in detectability. A third and final experiment replicated the well-known face inversion effect using the same set-up as Experiments 1 and 2, thereby demonstrating that the employed methodology can capture more high-level effects as well. These results are discussed in the context of previous evidence on unconscious semantic processing and two potential explanations are advanced. Specifically, it is argued that CFS might act at a level too low in the visual system for high-level effects to be observed or that the widely used breaking CFS paradigm is merely ill-suited to capture effects in the context of words.

## Introduction

Although our visual awareness of the world most of the time is continuous and stable, sometimes conscious perception fluctuates while retinal input stays constant. Amongst other, this situation arises when the two eyes are presented with different stimuli at corresponding retinal locations. Instead of mixing the signals of both eyes based on, for example, a weighted sum, the visual system appears to “decide” to categorically favor the image presented to one eye or the other and to stochastically alternate between the two interpretations, a phenomenon known as binocular rivalry. Since conscious perception fluctuates while visual input does not change, binocular rivalry has been proposed as a technique to study the (neural) correlates of consciousness [1]–. However, the stochastic nature of the rivalry process made it hard for researchers to reliably suppress stimuli for a time period that allowed them to measure the extent to which these suppressed stimuli were still processed in the absence of awareness. Continuous flash suppression (CFS) proved to be a solution to this problem by introducing a repetitive, dynamic signal in one eye which seemed to more effectively suppress the stimulus presented in the other eye [4], [5]. Upon its introduction, CFS was rapidly picked up on as a reliable technique to study unconscious processing of various classes of stimuli. One of these research lines pertained to whether semantic information of words is extracted in the absence of awareness. To study unconscious semantic processing of words, the breaking CFS paradigm (b-CFS, [6]) has been mostly used. In b-CFS, a stimulus is presented in one eye (usually at low contrast) and a CFS mask in the other. Due to its high contrast and dynamic nature, the CFS mask dominates initially. The contrast of the other stimulus is then gradually increased until it “breaks through” the CFS mask. The time until breakthrough (i.e., suppression time) is commonly used as an index of unconscious processing of the stimulus. That is, if different stimuli break CFS on average differentially, it is assumed that some kind of unconscious representation must have biased the breaking through CFS (e.g., see [7]).

Upon reviewing the literature, it became apparent that some conflicting findings had been reported with respect to the unconscious processing of semantic information of words. For example, Costello et al. [8] observed that suppression times of words were influenced by the prime-target relation of a previously presented visible prime word. That is, when a semantically related prime preceded the suppressed target, it broke suppression faster than when prime and target were unrelated. Seemingly in contrast, Sklar et al. [9] found that short semantically incongruent sentences broke suppression *faster* than semantic congruent sentences.

Another line of research pertains to the question whether semantic information of emotional words is extracted in the absence of awareness. To address this question, Yang and Yeh [10] presented participants with neutral and negatively valenced Chinese two-character words. They observed that negative words take longer to break suppression than neutral words. In apparent contradiction with the findings of Yang and Yeh [10], Sklar et al. [9] report on experiments in which a negatively valenced combination of two neutral words (e.g., black eye) broke suppression faster than a neutral combination of two neutral words.

In sum, no consistent pattern of findings has emerged from the studies on unconscious semantic processing of words. For semantic congruency relations as well as for negatively valenced words or word relations, studies disagree as to whether such stimuli break suppression slower or faster. It is noteworthy that these studies all addressed relatively specific questions regarding unconscious processing of *words*. However, it has not been clearly established that words indeed have a special status. That is, no study has yet probed whether a difference would be observed between suppression times of words and non/pseudo-words presented under CFS. Secondly, we sought to assess whether the word frequency effect, one of the most robust findings in the psycholinguistic literature (e.g., [11]), would manifest itself under CFS. That is, visual word recognition occurs faster for highly frequent words. Here, we investigated whether suppression times of words also correlate with their respective word frequency. In our first experiment, we set out to test both hypotheses. That is, we generated a set of words varying in word frequency and an associated set of pseudo-words. These stimuli were presented under CFS and participants had to indicate the position of the suppressed stimulus upon breakthrough (i.e., either below or above a fixation cross). To preview our results, we found no evidence for differential suppression times between words and pseudo-words nor a correlation between word frequency and suppression time.

## Experiment 1

### Materials and Methods

#### Ethics Statement

The study was conducted in line with the ethical principles regarding research with human participants as specified in The Code of Ethics of the World Medical Association (Declaration of Helsinki). The study was approved by the Ethical Committee of the Faculty of Psychology and Educational Sciences (EC FPPW) of the University of Leuven, and the participants gave written informed consent before starting the experiment.

#### Participants

Eighteen healthy subjects (6 male, age range 18–30 years) volunteered for the experiment and were paid for their participation. All participants had normal or corrected-to-normal vision and were naïve with respect to the goal of the study.

#### Apparatus

Stimuli were shown on two 19.8-in. Sony Trinitron GDM F500-R (2048×1536 pixels at 60 Hz, for each) monitors driven by a DELL Precision T3400 computer with an Intel Core Quad CPU Q9300 2.5 GHz processor running on Windows XP. Binocular presentation was achieved by a custom made stereo set-up. Two CRT monitors, which stood opposite to each other (distance of 220 cm), projected to the left and right eye respectively via two mirrors placed at a distance of 110 cm from the screen. A vertical plate ensured stable projection from the left and right screen to the left and right eye, respectively. A head- and chin rest (15 cm from the mirrors) were used to stabilize fixation. The effective viewing distance was thus 125 cm. Stimulus presentation, timing and keyboard responses were controlled with custom software programmed in Python 2.7 using the PsychoPy library [12], [13].

#### Stimuli

A random checkerboard pattern was used as the background display to achieve stable binocular fusion. The individual elements of the checkerboard subtended 0.34° by 0.34°. For stimulus presentation, two grey frames were superimposed on the checkerboard pattern (frame size 10° by 10°). A white fixation cross (0.6° by 0.6°) was continuously present during the experiment.

The main experiment was preceded by an eye dominance measurement phase in which the target was an arrow (maximal width 4°; maximal height 2°). For measuring eye dominance, the CFS mask consisted of 150 squares with a randomly picked size (between 1° and 2° width) and a random grayscale value on each refresh of the mask. In the main experiment, the size of the individual elements of the CFS mask ranged between 0.2° and 1.2°. The mask contained 200 squares with a randomly picked color on each refresh. In all the phases of the experiments reported here, the refresh rate of the CFS mask was set to 10 Hz.

A total of 154 Dutch words were selected from the SUBTLEX-NL database, which, as a whole, showed a word frequency effect on lexical decision latencies and accuracies [14]. Word frequency was operationalized as the log-transformed number of contexts in which a word occurs [15] and ranged from 0.669 to 3.882 (see Table 1 for a summary of the stimulus characteristics). The word stimuli were then used as input for Wuggy, a program that generates pronounceable pseudo-words [16]. Thus, each word had an orthographically similar pseudo-word counterpart (e.g., *lamp* – *hamp*). The size of the words ranged from 0.92° to 3.9° depending on the length of the word, which varied from two to seven letters. The height of the words was maximally 0.92°. In addition to word length, we also derived a measure of pixel density by summing all pixels comprising each stimulus. Furthermore, we obtained more high-level characteristics such as age of acquisition (i.e., an estimate of the age at which a word has been learned) and concreteness (i.e., an estimate of how concrete a concept is) from Moors et al. [17] and Brysbaert et al [18].

**Table 1:** Descriptive Statistics for the Words in Experiment 1 and 2.

Factor	Mean (*SD*s in parentheses) forExperiment 1	Mean (*SD*s in parentheses) forExperiment 2
Word Frequency	2.33 (0.74)	2.29 (0.76)
Word Length	4.22 (0.83)	4.57 (0.98)
Pixel Density	5,364 (1,130)	5,799 (1,468)
Age of Acquisition	7.39 (2.23)	7.37 (2.13)
Concreteness	4.14 (0.87)	4.11 (0.90)

*Note*. Word Frequency is the log-transformed number of contexts in which a certain word occurs [14]. Word Length is the number of characters. Pixel Density refers to the sum of all pixels that comprised the stimulus. Age of acquisition is the estimated age in years at which a word is learned [17], [18]. Concreteness is an estimate on a five-point likert scale of how concrete a concept is (the higher, the more concrete) [18]. Age of acquisition and concreteness estimates were not available for one word in both Experiment 1 and 2.

#### Procedure

Prior to the start of the main experiment, participants’ eye dominance was measured according to the method of Yang et al. [19]. On every trial, participants were presented with an arrow in one eye gradually increasing contrast from 0 to 100% and pointing either left or rightwards. In the other eye, the CFS mask was presented. As soon as the arrow broke suppression, participants had to indicate its direction by pressing 1 or 3 on the numerical keyboard for the left and right direction, respectively. Subsequently, eye dominance was determined by comparing the average suppression times of the left eye to that of the right. The eye for which the average suppression time was the lowest was considered to be the dominant eye. Consequently, the CFS mask was presented in this eye throughout the rest of the experiment.

**Figure 1:**
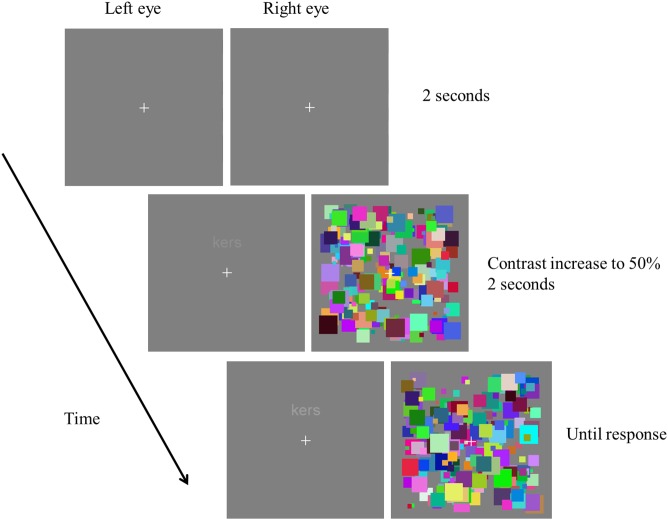
Example of the trial sequence. First, a fixation cross was presented for 2 seconds. Subsequently, the CFS mask was presented in one eye and the (non/pseudo)-word stimulus in the other. The (non/pseudo)-word stimulus increased in contrast from 0 to 50% over the course of 2 seconds and was continuously present until participants made a response.

In the main experiment, the word or pseudo-word stimuli were presented in lower case letters either 2° above or below the fixation cross and gradually faded in from 0 to 50% contrast over a period of 2 seconds (see Figure 1). Upon breakthrough, participants had to indicate as fast as possible the location of the stimulus (above or below fixation) with a button press on the numerical keyboard (1 for above, 3 for below), initiating a new trial. A fixation cross was presented during the intertrial interval, which lasted 2 seconds.

#### Design

During the eye dominance measurement phase, participants completed 80 trials in total, in half of which the target was presented to the left eye. For each eye, half of the targets pointed leftwards. Trial presentation was randomized.

The main experiment consisted of 308 trials, 154 word trials and 154 pseudo-word trials, split up in two blocks between which participants took a break of at least one minute. Besides the word type manipulation, we also used word stimuli that varied in frequency of occurrence. To ensure that one element of a word - pseudo-word pair (e.g., *lamp*) could not prime the other (e.g., *hamp*), they were always presented in different blocks. That is, the words of a random half of the pairs were presented in the first block together with the pseudo-words of the second half of the pairs and vice versa for the second block. Block order was counterbalanced across participants through their participant number (odd or even). Presentation order of the stimuli within a single block was randomized for each participant. The position of the stimuli was randomized, such that half of the stimuli appeared above the fixation cross and the other half below. Word – pseudo-word pairs were linked in the sense that they either appeared both above or below fixation. Furthermore, stimuli presented above and below fixation were matched in terms of word frequency (*M*_above_ = 2.33, *M*_below_ = 2.33, Bayes Factor = 6). Position was kept constant across participants. Prior to the start of the main experiment, participants completed 20 different practice trials to familiarize themselves with the task.

### Results and Discussion

All analyses were done on correct trials only (1.9% of the data had to be removed). Furthermore, data points below 500 ms or more than three standard deviations above each participant’s mean suppression time were not included in the analysis (1.5% of the correct trials). Suppression times were log-transformed due to their positive skewness. All analyses were conducted within the Bayesian statistical framework using the BayesFactor package to calculate Bayes Factors (BF) and 95% credible intervals [20], [21]. In contrast to classical null hypothesis testing, a Bayesian approach allows to quantify evidence in favor of either the null or the alternative hypothesis [20]–[22]. All models tested here are so called mixed models as they consist of both fixed and random effects. The random part of the models was kept constant across all analyses and included random intercepts for participants and for words. To facilitate the interpretation of the results, we always z-transformed continuous variables and we also report t-statistics and 95% confidence intervals for the same models using the lme4 package [23] (see Supplementary Table S1).

**Figure 2:**
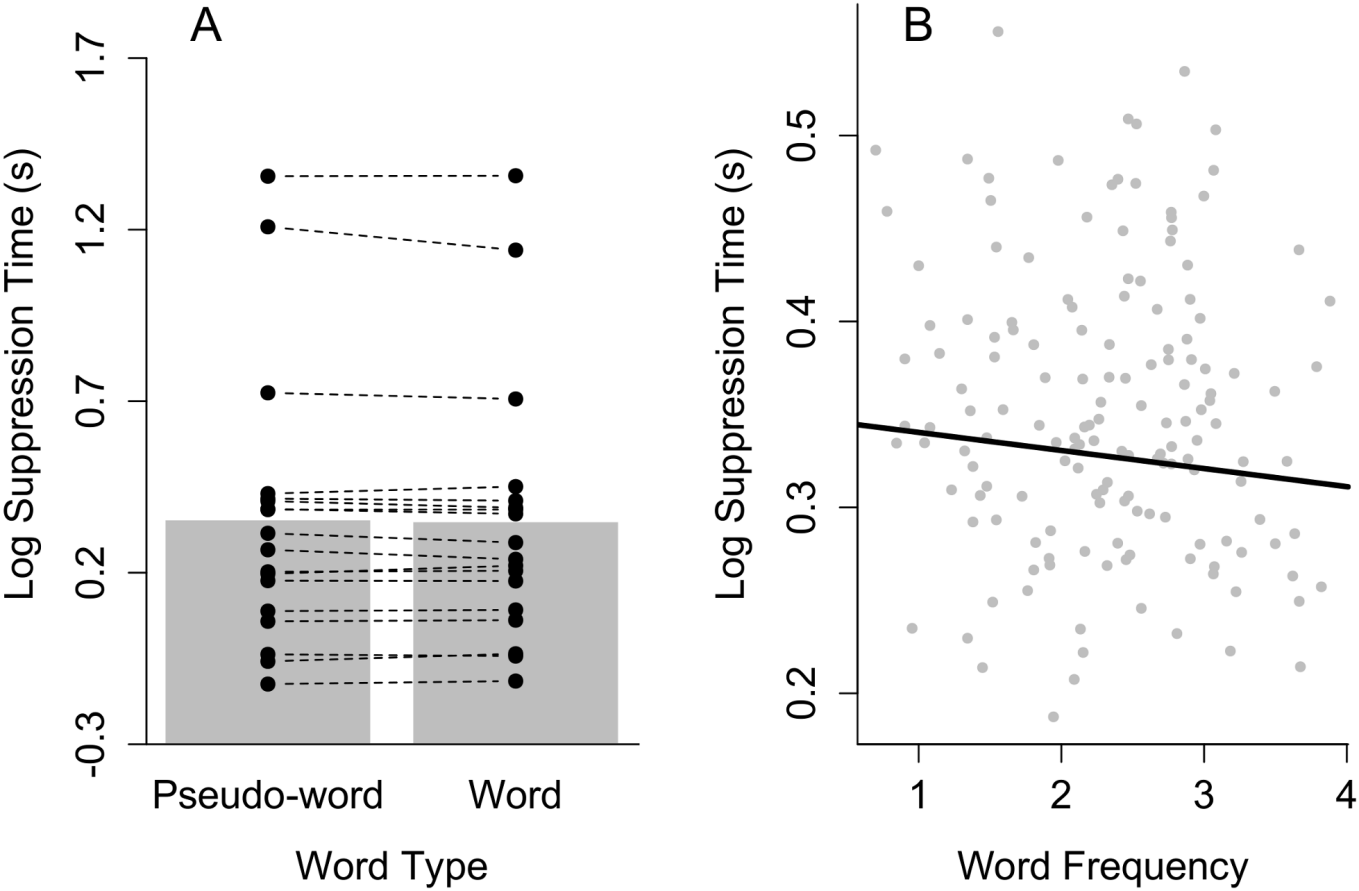
Results of Experiment 1. (A) The bar plot indicates mean log suppression times for words and pseudo-words. The dots show the mean log suppression time for each participant (connected dots refer to the same participant). (B) Scatter plot depicting the (absence of a) relationship between word frequency and log suppression time. The data points refer to mean log suppression time for each item averaged across participants. The black line refers to the posterior estimate of the relationship between word frequency and log suppression time based on a mixed-effects model fit (with the BayesFactor package) with participants and words as crossed random effects and word frequency as a fixed effect.

Figure 2 depicts average log suppression times for words and pseudo-words together with individual data points (left) and the relationship between word frequency and suppression time (right) (see Figure S1 for untransformed suppression times). Through eye balling the results it already becomes clear that there is neither an effect of word type nor word frequency on suppression time. This was confirmed in the BF analysis (see Table 2 for estimates of the fixed effects). The BF for a model including the effect of word type and random intercepts for participants and words was not favored over the random effects only model (BF = 26, i.e., the random effects only model was 26 times more likely). Similar results were obtained for the word frequency data (BF = 11). Both analyses were run separately since there was no meaningful value for word frequency of pseudo-words.

**Table 2:** Point Estimates and 95% Credible Intervals of the Fixed Effects in Experiment 1 and 2.

Model	Experiment 1		Experiment 2	
	Estimate	95% CI	Estimate	95% CI
**(1)**				
*Mu*	0.354	[0.164; 0.557]	0.330	[0.226; 0.435]
*Pseudo/Non - word*	0.003	[−0.005; 0.010]	0.003	[−0.0006; 0.007]
*Word*	−0.003	[−0.010; 0.005]	−0.003	[−0.007; 0.0006]
*Inverted*	NA	NA	0.002	[−0.002; 0.006]
*Upright*	NA	NA	−0.002	[−0.006; 0.002]
**(2)**				
*Mu*	0.350	[0.160; 0.536]	0.330	[0.221; 0.435]
*Word Frequency*	−0.007	[−0.022; 0.007]	−0.007	[−0.020; 0.006]
*Inverted*	NA	NA	0.001	[−0.004; 0.007]
*Upright*	NA	NA	−0.001	[−0.007; 0.004]
**(3)**				
*Mu*	0.350	[0.161; 0.538]	0.330	[0.220; 0.434]
*Word Frequency*	−0.012	[−0.032; 0.009]	−0.003	[−0.020; 0.013]
*Inverted*	NA	NA	0.001	[−0.004; 0.007]
*Upright*	NA	NA	−0.001	[−0.007; 0.004]
*Pixel Density*	−0.016	[−0.030; −0.002]	−0.036	[−0.049; −0.025]
*Trial*	−0.053	[−0.064; −0.042]	−0.051	[−0.057; −0.046]
*Age of Acquisition*	−0.008	[−0.029; 0.011]	−0.004	[−0.019; 0.011]
*Concreteness*	−0.008	[−0.025; 0.009]	−0.005	[−0.018; 0.009]
**(4)**				
*Mu*	0.352	[0.162; 0.547]	0.332	[0.227; 0.437]
*Pixel Density*	−0.019	[−0.030; −0.009]	−0.035	[−0.046; −0.025]
*Trial*	−0.060	[−0.067; −0.052]	−0.052	[−0.056; −0.048]

*Note.* Per experiment, the parameter estimates of the fixed effects of four models are reported. Model (1) comprised only the main effect of word type (and of inversion in Experiment 2). Model (2) tested the main effect of word frequency (and inversion in Experiment 2). Model (3) is an expansion of Model (2) in that the main effects of pixel density, trial number, age of acquisition and concreteness were added. Finally, model (4) only consists of the main effects of pixel density and trial number. Models (1) and (4) were fitted using all data, model (2) was run on the word data only and model (3) included all words except one because concreteness and age of acquisition estimates were not available for this stimulus. To facilitate the comparison, all continuous variables were z-transformed (see Table 1 for means and standard deviations of the variables).

In addition, it is known that word frequency correlates with many other sublexical, lexical and semantic variables [24], [25]. Hence, it is possible that the true word frequency effect was masked in the previous analysis. To test this hypothesis, a supplementary analysis was conducted in which a number of covariates were added to isolate the “pure” word frequency effect. That is, besides word frequency, we included main effects of age of acquisition, concreteness, pixel density and trial number. As age of acquisition and concreteness data were unavailable for one word, the analysis was performed on the remaining 153 words. Word length was left out to avoid potential multicollinearity issues as it correlated highly with pixel density (*r* = .80).

The estimates of the fixed effects (see Table 2) seem to suggest that neither concreteness, age of acquisition nor word frequency are related to suppression time as their corresponding 95% credible intervals all include zero. Trial number and pixel density on the other hand, do seem to have an influence, in that suppression times became faster as the experiment advanced and as words contained more pixels. The obtained BFs confirm these findings (see Table 3). Two models are equally preferable, one with trial as only predictor and one with both trial and pixel density as predictors. All other models are at least eight times less likely.

**Table 3:** Bayes Factors for the Additional Analysis of Experiment 1.

Model	Bayes Factor
Trial	1
Pixel Density + Trial	1
Word Frequency + Pixel Density + Trial	8
Word Frequency + Trial	9
Pixel Density + Trial + Concreteness	10
Pixel Density + Trial + Age of Acquisition	10
Trial + Age of Acquisition	12
Trial + Concreteness	12
Word Frequency + Pixel Density + Trial + Concreteness	59
Word Frequency + Pixel Density + Trial + Age of Acquisition	62
Word Frequency + Trial + Concreteness	72
Word Frequency + Trial + Age of Acquisition	78
Pixel Density + Trial + Age of Acquisition + Concreteness	82
All other models	>100

*Note.* The Bayes Factor is relative to the model with trial number as only predictor and random intercepts for subjects and words. A Bayes Factor >1 indicates evidence for the trial number only model. Models are ordered from low to high in terms of their Bayes Factor.

To further examine the effects of trial and pixel density, an additional analysis was run on both words and pseudo-words using only these two variables (see Table 2). The results are very similar, except that the model with both trial and pixel density was now clearly preferred over a trial only model (BF = 33), a pixel density only model (BF>100) and a null model (BF>100).

In Experiment 1, a set of word stimuli varying in word frequency and word type (word vs. pseudo-word) were presented under CFS and participants had to detect, upon breakthrough, as fast as possible whether the word stimulus was presented either above or below fixation. It was hypothesized that, given that semantic information of word stimuli is extracted in the absence of awareness, more frequent words would break suppression faster and words would break through suppression faster than pseudo-words. Contrary to our predictions, we found neither an effect of word frequency nor of word type. In additional analyses, we did however find a trial effect indicating that suppression times shortened over the course of the experiment (see [26] for similar observations). We interpret this trial effect as indicating that participants did not press randomly across the experiment, but were engaged in the task until the end. Moreover, pixel density of the stimuli also predicted suppression in that stimuli that comprised fewer pixels had longer response times. A similar effect was found by Lupyan and Ward [27] using pictures as stimuli, which was taken to mean that the effectiveness of suppression depends on stimulus-driven factors like signal strength.

Although the evidence for a null effect in Experiment 1 was quite strong (according to the criteria advanced by Jeffreys [28]), alternative explanations can be devised as to why a null effect would be observed. First, the pseudo-words used in Experiment 1 were still word-like in the sense that they were pronounceable and orthographically similar to existing words. Thus, these pseudo-words might have activated the semantic network to an extent comparable to real words yielding no suppression time difference between words and pseudo-words. Therefore, in Experiment 2, non-words were generated by randomly jittering the individual letters of the words (e.g., *lamp* resulted in *mlap*). Second, one could argue that, although semantic information of words might not be processed, familiarity of the individual letters still is. Indeed, the potential role of stimulus familiarity (of the individual letter) cannot be disentangled from the design of Experiment 1. Therefore, we included a condition in Experiment 2 in which we presented the words and non-words inverted to assess the role of familiarity in breaking suppression [29], [30]. Third, a potential criticism of Experiment 1 could be that our mask was just not sensitive enough to detect any difference between our conditions. It should be noted though, that this explanation is at odds with the observed pixel density effect. That is, suppression appeared to be stronger when the bottom-up signal was relatively weak (see also Lupyan and Ward [27]). Nevertheless, we addressed this in Experiment 2 by including a control experiment in which, instead of a word stimulus, a simpler stimulus (a white disc) was presented under CFS. The size of this disc was varied and it was hypothesized that the smaller disc would break suppression slower on average than the bigger disc, if suppression effectively takes place. Fourth, we observed that the consistency over participants in suppression time was rather low (i.e., Cronbach’s α = .20). Put differently, there was no stability across participants in which words broke suppression early and which words were relatively delayed. To further examine this issue, Experiment 2 consisted of a test-retest design such that it was possible to evaluate whether suppression time is stable *within* participants.

## Experiment 2

### Materials and Methods

#### Participants

Twenty new paid participants (4 male, age range 18–30 years) were recruited for Experiment 2. All participants had normal or corrected-to-normal vision and were naïve with respect to the goal of the study. Every participant provided informed consent before the start of the experiment. Note that, due to a programming error for participants with odd subject numbers, we had to rerun our original sample of 20 participants with 10 new participants with an odd subject number, but keeping the original participants with an even subject number. Furthermore, 4 participants were not included because they did not complete the full experiment. One of them did not show up for the retest session, the others did not finish the first session due to suppression being too effective.

#### Apparatus

The experimental set-up was the same as in Experiment 1.

#### Stimuli

All stimuli were the same as in Experiment 1 except for the following. A partially new set of word stimuli was created to ensure that the findings from Experiment 1 could not be attributed to the specific stimulus set used (see Table 1 for a summary of the stimulus characteristics). Word length varied from three to seven letters and word frequency from 0.669 to 3.882. There were 115 words in total, from which unpronounceable non-words were created by shuffling the letters. To test the effect of stimulus familiarity, the 230 words and non-words were inverted, thus yielding 460 stimuli in total. The size of the words ranged from 1.56° to 4.35° depending on the length of the word. The height of the words was maximally 0.92°.

In the control experiment, a white disc was presented as a target instead of a word. The radius of the disc was manipulated to be either 0.6° or 1.2°.

#### Procedure

The experimental procedure was similar to Experiment 1. Prior to the start of the main experiment, participants completed the eye dominance experiment. The task in the main experiment was exactly the same as in Experiment 1. In the control experiment, a white disc increasing in contrast from 0 to 100% over the course of 2 seconds was presented either 2° above or below fixation. As in the main experiment, participant had to indicate the location of the disc as fast as possible once it broke suppression through a button press on the numerical keyboard (1 for above, 3 for below). A second session always took place 24 hours after the first session and included only the main and control experiment.

#### Design

The main independent variables were word type (word vs. non-word), inversion (upright vs. inverted) and word frequency (ranging from 0.669 to 3.882). In the control experiment, disc radius was manipulated (small vs. large; 0.6° vs. 1.2°). Before the start of the main experiment, participants again performed 20 practice trials on a different set of stimuli. The main experiment now consisted of 460 trials (i.e., 115 words, 115 non-words and their inverted counterparts) and therefore was split up into four blocks. Similar to Experiment 1, the words of a random half of the word – non-word pairs were presented in the first half of the experiment together with the non-words of the second half of the pairs and vice versa. The position of the stimuli was again determined at random and kept constant across participants. Similar to Experiment 1, word – non-word pairs and their inverted counterparts were all either presented above or below fixation. As a result the number of stimuli appearing above and below fixation was not perfectly identical (i.e., 232 stimuli below fixation and 228 above). Stimuli were again matched on word frequency (*M*_above_ = 2.24, *M*_below_ = 2.34, BF = 4).

After completing the main experiment, the experimenter started the control experiment in which participants had to detect a white disc that was either presented 2° above or below fixation. They first completed 20 practice trials and subsequently performed 100 trials in the control experiment (50 per condition, randomized on each trial). On the second day, participants returned to perform the experiments in the same order again, except for the eye dominance measurement which was not repeated.

### Results and Discussion

#### Main experiment

**Figure 3:**
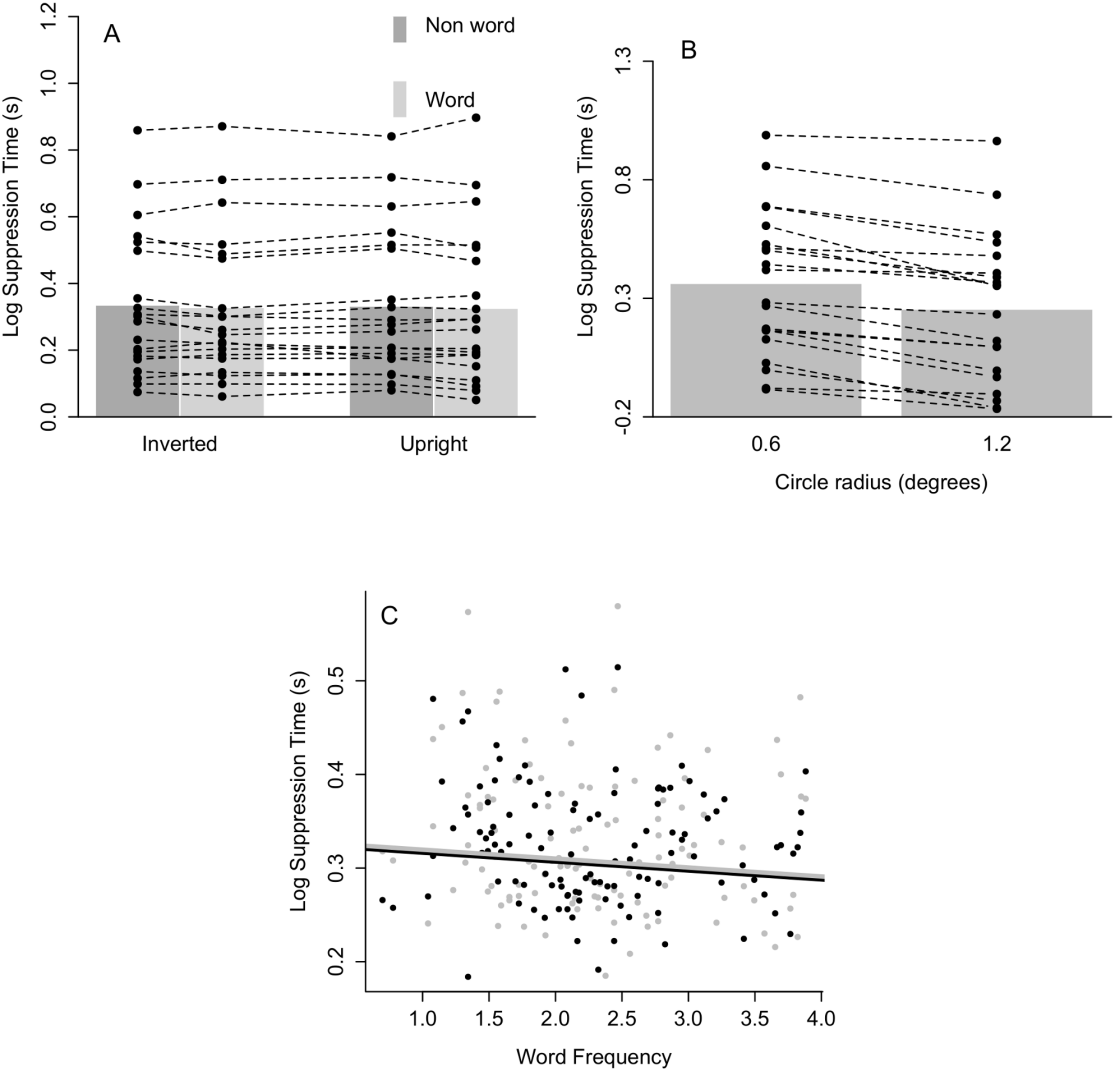
Results of Experiment 2. (A) The bar plot depicts the mean log suppression times for each condition. The dots refer to mean log suppression times per participant (connected dots refer to the same participant). (B) Mean log suppression times for the control experiment. The bar plot depicts the grand mean for both conditions whereas the dots refer to single participants (connected dots refer to the same participant). (C) Scatter plot depicting the (absence of a) relationship between word frequency and log suppression times for upright (black) and inverted (gray) words. The black and gray lines (hardly discernible) refer to the estimates of the relationship between word frequency and log suppression time after a mixed-effects model fit with subject and word as crossed random effects and word frequency and inversion as fixed effects.

Figure 3 summarizes the results of Experiment 2 (see Figure S2 for untransformed suppression times). As in Experiment 1, analyses were done on the logarithmically transformed suppression times after removal of inaccurate responses (1.6% of all data) and outlying data points (defined as being below 500 ms or higher than each participant’s mean suppression times plus three times the standard deviation; 1.5% of all correct trials). Again, all models fitted here are mixed models with random intercepts for participants and for words.

The results shown in Table 4 indicate that neither an effect of word type nor inversion nor an interaction between both is present in the data (see also Table 2 for the parameter estimates of the model including only the main effects of word type and inversion). In addition, an analysis on only the word stimuli did not reveal an effect of word frequency, inversion or an interaction between both variables (see Table 5 for Bayes Factors; and Table 2 for parameter estimates of the main effects only model). Taken together, the (empty) random intercepts only model was always preferred.

**Table 4:** Bayes Factors for the Analysis of Word Type and Inversion of Experiment 2.

Model	Bayes Factor
Random Intercepts Only	1
Word Type	15
Inversion	45
Word Type + Inversion	>100
Word Type * Inversion	>100

*Note.* The Bayes Factor is relative to the null model, including only random intercepts for subjects and words. A Bayes Factor >1 indicates evidence for the null model.

**Table 5:** Bayes Factors for the Analysis of Word Frequency and Inversion of Experiment 2.

Model	Bayes Factor
Random Intercepts Only	1
Word Frequency	10
Inversion	36
Word Frequency + Inversion	>100
Word Frequency * Inversion	>100

*Note.* The Bayes Factor is relative to the null model, including only random intercepts for subjects and words. A Bayes Factor >1 indicates evidence for the null model.

As in Experiment 1, we ran an additional analysis to statistically control for confounding variables (i.e., concreteness, age of acquisition, pixel density and trial number). The results replicate our previous findings in that the model with trial number and pixel density was preferred over all other models by a factor of at least ten (see Table 6). Also, when looking at the model with the main effects of word frequency, inversion, concreteness, age of acquisition, pixel density and trial number, it can be seen that only the 95% credible intervals of trial and pixel density exclude zero (see Table 2). The effects of trial and pixel density were confirmed in an additional analysis on both words and non-words using only these two predictors (see Table 2). That is, the model with both trial and pixel density was the best fitting model (all BFs >100).

**Table 6:** Bayes Factors for the Additional Analysis of Experiment 2.

Model	Bayes Factor
Pixel Density + Trial	1
Pixel Density + Trial + Concreteness	10
Pixel Density + Trial + Age of Acquisition	12
Word Frequency + Pixel Density + Trial	12
Word Inversion + Pixel Density + Trial	36
Pixel Density + Trial + Age of Acquisition + Concreteness	97
All other models	>100

*Note.* The Bayes Factor is relative to the model with trial number and pixel density as predictors and random intercepts for subjects and words. A Bayes Factor >1 indicates evidence for the trial number and pixel density only model. Models are ordered from low to high in terms of their Bayes Factor.

#### Control experiment

As is apparent from Figure 3, the data from the control experiment indicate an effect in the predicted direction. Concretely, the large disc broke through suppression faster than the small disc. This was confirmed by a Bayes factor (BF>100). The model including circle radius as a fixed effect and random subject intercepts was preferred over the random intercepts only model. The null effects observed in the main experiment can therefore not be attributed to a general lack of suppression elicited by our CFS mask.

#### Test-retest reliability

**Figure 4:**
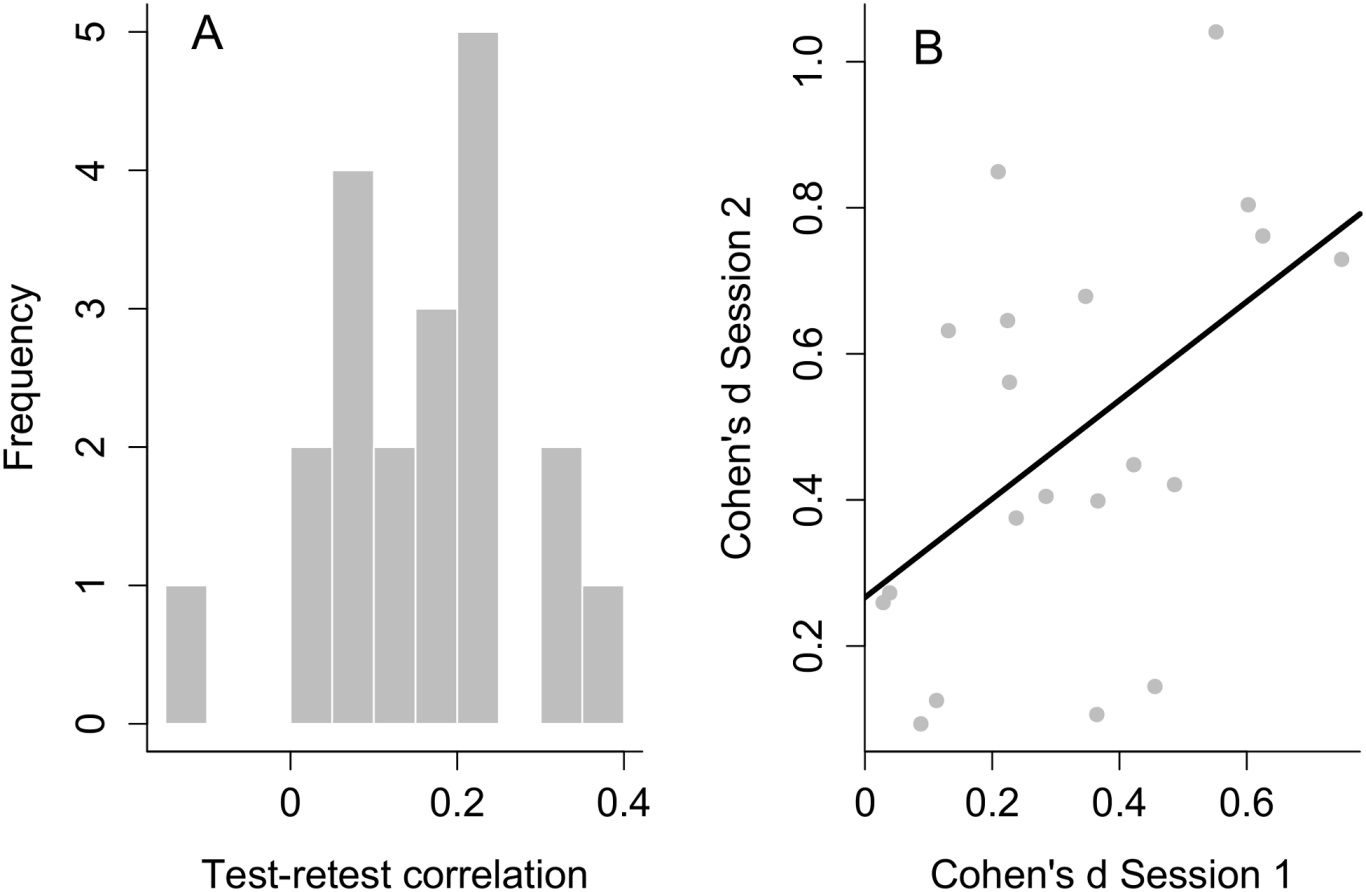
Test-retest reliability. (A) Histogram of test-retest correlations for every participant. (B) Scatterplot between the effect sizes obtained in sessions 1 and 2 of the control experiment. The black line refers to the best fitting regression line obtained from a simple linear regression of session 2 effect sizes on session 1 effects sizes.

Figure 4 depicts a histogram of the test-retest reliability scores for each participant in the main experiment. These correlations were computed by correlating the log suppression times for all 460 stimuli obtained in session 1 with those obtained in session 2. The mean test-retest reliability score was equal to .16 (ranging from −.10 to .37). Note that recalculating the test-retest reliability for the word stimuli only did not improve these correlations (mean .16, range from −.12 to .38). The right panel of Figure 4 depicts the test-retest reliability for the control experiment. Since this experiment only included repetitions of the same two stimuli, the effect size (Cohen’s d) for circle radius was computed for each participant on each session and correlated between sessions, yielding a correlation of .51.

In Experiment 2, some potential alternative explanations for the absence of a suppression time difference between words and pseudo-words were explored. First, the pseudo-words used in Experiment 1 might still have elicited some partial semantic activation, obscuring an effect of unconscious processing of semantic information. Therefore, in Experiment 2 unpronounceable non-words were used, generated by scrambling the individual letters of each word stimulus. Still, no evidence for an effect of word type was obtained. Secondly, stimulus familiarity might have contributed to the suppression times for each condition in Experiment 1 instead of semantic processing. Therefore, we included a condition in which the words and non-words were inverted, to examine the effect of stimulus familiarity while keeping low-level characteristics of the stimulus constant. Surprisingly, no evidence of an inversion effect was obtained, contrary to previous findings [10], [31]. In hindsight, the absence of an inversion effect is not that surprising given that inverting letters in the Latin alphabet does not always have a disruptive effect. That is, five letters remain the same when inverted (i.e., l, o, s, x, and z), six become another letter (i.e., b, d, n, p, q, and u), and some remain letter-like (e.g., m and w).

Third, the results of Experiment 1 showed low consistency across participants in suppression times. Therefore, Experiment 2 employed a test-retest design to further probe the reliability in both the main and control experiment. Test-retest reliability in the main experiment was on average rather low, indicating that there is considerable instability in the suppression times *within* participants. In the control experiment, the test-retest correlation approximated the estimate reported in Yang et al. [19] in which a similar measure was correlated across sessions. Although the latter correlation was still far from perfect, its comparability with the correlation reported in Yang et al. [19] speculatively hints at a potential ceiling for correlations of effect sizes based on stimulus manipulations in the CFS paradigm. Note that this does not mean that the data from the main experiment have no structure whatsoever. That is, some subjects showed a position bias for stimuli either presented above or below fixation and these effects correlated well across sessions (test-retest correlation for Cohen’s d of the position effect in the main experiment was .87).

Finally, the results of Experiment 1 could have potentially been explained by a lack of suppression initiated by the CFS masks or by a general insensitivity to detect any effect. To address this issue, a control experiment was conducted in which a simple stimulus, a white disc, was varied in radius. It was predicted that a large disc would break suppression faster than a small disc and the results of the control experiment confirmed this prediction. However, both the radius effect in circles and the pixel density effect in letter strings are fairly low-level. In principle it is possible that the present set-up is merely not sensitive enough to capture any high-level effect. That is, the lack of a word inversion effect could indicate a general lack of obtaining inversion effects using our implementation of b-CFS. To address this issue we set out to replicate the widely reported face inversion effect, in which faces presented upright break suppression faster than inverted faces [6], [7], [29], [30], [32]–[36]. In Experiment 3 the same b-CFS set-up was used, but the suppressed stimuli were (inverted or upright) faces instead of letter strings. If our b-CFS design is indeed unable to obtain high-level effects, one would expect no face inversion effect. Alternatively, finding a robust face inversion effect in light of the results of Experiments 1 and 2, would suggest that word frequency, word type and letter inversion have genuinely no effect on suppression times.

## Experiment 3

### Materials and Methods

#### Participants

Eight volunteers participated in the experiment (3 male, age range 24–34 years). All had normal or corrected-to-normal vision and were naïve with respect to the goal of the study. Every participant provided informed consent before the start of the experiment.

#### Apparatus

The experimental set-up was the same as in Experiments 1 and 2.

#### Stimuli

The same CFS mask was used as in Experiments 1 and 2. The face stimuli were obtained from the NimStim database [37]. Ten neutral faces were picked from the database (five male). These were resized to approximately 2.1°×2.6° (similar to Stein et al. [6]). Four different neutral faces (two male) were used for the practice trials.

#### Procedure

The experimental procedure was similar to Experiments 1 and 2. A trial started with a 2 second presentation of the fixation cross after which the CFS mask was presented in the dominant eye and the upright or inverted face stimulus in the non-dominant eye. As in Stein et al. [6], the face stimulus was presented at a random location to the left or right of fixation. The participants were instructed to report as quickly as possible the location of the stimulus (left or right relative to fixation, through a button press) upon the moment it broke suppression.

#### Design

The only independent variable was inversion (upright vs. inverted). Prior to the start of the main experiment, participants completed 16 practice trials to familiarize themselves with the task. During the main experiment, participants completed 120 trials in three blocks of 40 trials. For each participant, all ten faces were presented equally often in the inverted as in the upright condition and they were shown right of fixation in half of the trials and left in the other half. The order of the trials was randomized.

### Results and Discussion

**Figure 5:**
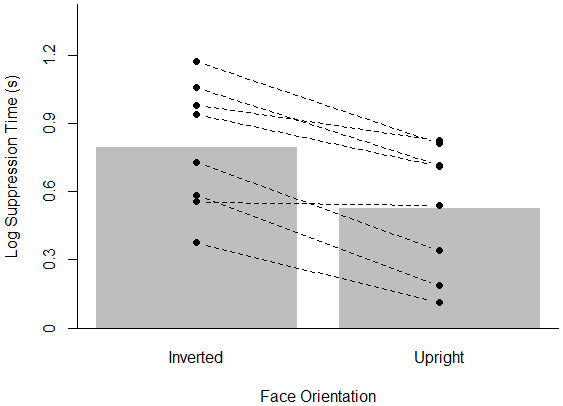
Results of Experiment 3. The bar plot indicates mean log suppression times for upright and inverted faces. The dots show the mean log suppression time for each participant (connected dots refer to the same participant).

As in Experiments 1 and 2, all reported analyses were done on the logarithmically transformed response times after removal of inaccurate (1.5%) and outlying data points (defined as below 500 ms or higher than each participant’s mean suppression time plus three times the standard deviation; 1.6% of all correct trials). Figure 5 summarizes the results of Experiment 3 (see Figure S3 for untransformed suppression times). There appears to be a strong inversion effect in that upright faces break through suppression faster than inverted faces. This was confirmed by comparing the model with face inversion as a factor against an empty model (both models also included random intercepts for participants and for faces). Specifically, the Bayes Factor indicated a clear preference for the model including face orientation over the empty model (BF>100). Furthermore, the 95% credible interval did not include zero (95% CI: [0.09; 0.18]).

Experiment 3 clearly replicated the face inversion effect, one of the most robust findings in the b-CFS literature, using the same set-up as in Experiments 1 and 2. Indeed, the absence of an inversion effect obtained in Experiment 2 could be due to a general lack of obtaining any kind of familiarity effect using our design (in contrast to our explanation of inverting individual letters of the Latin alphabet not effectively disrupting familiarity). The present results rule out the possibility that our b-CFS implementation disrupted any sensitivity to find inversion effects. Thus, it is not the case that the employed set-up did not allow us to detect high-level effects. This suggests that the findings of Experiments 1 and 2 genuinely reflect that more high level characteristics such as word frequency, word type (words vs. non-words) and letter inversion do not influence suppression times.

## General Discussion

The goal of this study was to explore two hypotheses regarding unconscious processing of semantic information of words presented under CFS. First, it was predicted that existing words would break suppression faster than their pseudo-word/non-word variants. Second, we tested whether the suppression time of words is modulated by their frequency, resembling the word frequency effect in visual word recognition. Across two experiments, we found neither a word type effect nor a frequency effect. While the lack of a word type effect in Experiment 1 could be attributed to the use of pronounceable pseudo-words as a baseline, Experiment 2 excluded this explanation, as words did not break suppression faster than unpronounceable non-words. In addition, the fact that there was a consistent negative relation between pixel density and suppression time, suggests that the observed null results can not be attributed to the paradigm being insensitive to differences in detectability. The latter was further supported by Experiment 3, which showed that upright faces broke suppression faster than inverted ones using the exact same b-CFS set-up. Thus, even though the employed paradigm can capture high-level effects, only variability in low-level word characteristics like pixel density led to differential suppression times. Taken together, our findings do not support the claim that words are processed up to a semantic level under CFS.

In the visual masking literature on the other hand, unconscious semantic processing has been established [38], [39]. Should there be any reason to expect differences between visual masking and CFS paradigms? Discrepancies between unconscious processing of emotional information of faces have been reported in the context of masking, interocular suppression, and gaze-contingent crowding [40]. In visual masking, one explanation as to why the masked stimulus does not enter visual awareness is that re-entrant activation from higher cortical areas, presumably associated with perceiving the stimulus [41], [42], is nearly absent, yet the feed-forward sweep of activation associated with presentation of the masked stimulus is largely intact [43], [44]. CFS, however, relies on binocular rivalry of which the suppression mechanisms have mostly been attributed to inhibition between monocular neurons, although most recent models of binocular rivalry indicate potential inhibition mechanisms between higher levels of the visual system also [1], [3], [45]. Indeed, neuroimaging studies have indicated that processing of suppressed stimuli beyond striate areas is largely absent along the ventral visual pathway [46], [47]. As a consequence, any processing of semantic information under CFS seems implausible. Indeed, in a standard dissociation study using CFS, Kang et al. [48] explicitly showed that parametrically manipulating target visibility attenuated the amplitude of the N400 component (an index of semantic congruency) until it was absent when observers could not discriminate the meaning of the suppressed words.

An alternative explanation of the present findings is that semantic information is indeed extracted under CFS, but that the b-CFS paradigm is ill-suited to unambiguously detect these effects. That is, our results showed that suppression times are unstable both between and within participants. Such a poor reliability has rather dramatic effects on the probability of detecting a true underlying relation. An average test-retest reliability of .16 as observed in Experiment 2 could attenuate a true correlation of, say .60, to .24 (note that this example only considers the reliability of one variable, in this case suppression time, thereby (unrealistically) assuming that the other measure (e.g., word frequency) is perfectly reliable. In practice, the .24 estimate may thus even prove to be too optimistic). So even if there actually is a relation between suppression breaking and word frequency, it might go undetected using this paradigm. In comparison, reliability estimates of (log-transformed) response times in traditional word recognition studies generally range from .70 to .90 (e.g., [11], [49]). However, the low reliability observed here is specific to our stimuli and does not need to generalize to other stimuli like pictures or the b-CFS paradigm in general.

Furthermore, it should be noted that criticisms have been raised concerning the validity of the b-CFS paradigm to infer unconscious processing of suppressed stimuli [6], [50]. That is, the dependent measure used in b-CFS studies is the time it takes for subjects to be able to make a response on a certain attribute of the suppressed stimulus (e.g. its location). This suppression time measure per se is based on *conscious* processing. However, the argument to use b-CFS as a valid way to infer unconscious processing is that differences in suppression times are attributable to unconscious processing of the stimulus while suppressed. For this reasoning to be valid, the observed suppression time differences should be due to CFS-specific processing and not non CFS-specific threshold differences. To rule out this possibility, Jiang et al. [7] and subsequent studies usually implemented a binocular control condition in which the CFS mask and stimulus are simultaneously presented in both eyes. However, Stein et al. [6] have recently shown that this control condition is ill-suited to exclude non CFS-specific processing in the CFS condition since both conditions differ on aspects other than CFS-specific processing. Based on these findings, Stein and Sterzer [50] recently argued that b-CFS, as it is currently implemented, can not unequivocally provide evidence for unconscious processing of the suppressed stimulus.

Taken together, the criticisms raised by Stein and colleagues [6], [50] and our low reliability estimates seem to imply that the use of b-CFS as a paradigm to study unconscious semantic processing of words is questionable. Hence, we would argue that other paradigms combined with CFS might be more appropriate to probe the nature of processing of suppressed words (see also [51]). For example, it might be valuable to present suppressed words as primes and to study their influence on the reaction times to (un)related targets in, for example, a lexical decision task. Nevertheless, the question remains as to which mechanisms underlie the (seemingly contradicting) effects observed in the literature. Below, we offer some speculative explanations, but it should be noted that future research and/or re-analysis of existing datasets is needed to assess their validity.

One possibility is that familiar stimuli break suppression faster than unfamiliar stimuli [29], [30]. Such a familiarity effect has been observed by Jiang et al. [7]. In one of their experiments, Chinese and Hebrew speakers were presented with Chinese and Hebrew words under CFS. Jiang et al. [7] observed that Chinese words broke suppression faster for Chinese speakers as well as Hebrew words for Hebrew speakers. Furthermore, Yang and Yeh [10] also examined familiarity effects by comparing upright words with inverted and phase-scrambled words. Both inverting and phase-scrambling the character words significantly increased suppression times relative to upright words. These findings together with those of Jiang et al. [7] do provide evidence for a potential familiarity effect under b-CFS. In contrast, we did not obtain an inversion effect in Experiment 2, but in hindsight this is not entirely unexpected if individual characters are the locus of the familiarity effect. Specifically, inverting Latin letters often yields the same letter (e.g., *o*) or a different letter (e.g., *d* becomes *p* and vice versa), thus yielding (partially) familiar character strings. In addition, research shows that an inversion effect is not ubiquitous. For instance, Stein et al. [29] found an inversion effect of human faces and bodies, but not of inanimate objects.

Note that a familiarity effect could be the result of bottom-up processes (i.e., unconscious processing occurs to a certain extent under CFS and familiar stimuli, or familiar parts, break suppression faster) as well as top-down processes (i.e., subjects generate familiar representations that are matched with the visual input, which in turn facilitates suppression breaking). The latter mechanism could also explain the priming effect found by Costello et al. [8]. Presumably, subjects generate a set of candidate targets based on the prime (e.g., *dog*, *pet*, *animal* when the prime is *cat*). The visual representation of these candidates might boost the detection of the actual target, when prime and target are indeed related, through a matching process. In a recent study by Lupyan and Ward [27], a similar biasing effect has been reported in that informative verbal labels (presented auditorily) biased detection performance of suppressed visual stimuli relative to uninformative verbal labels. This effect was attributed to top-down activation of the visual shape properties of the suppressed stimuli which eventually biased the competition process [27]. Note that such a top-down process implies that, for example in the study of Costello et al. [8], semantic processing of the suppressed stimulus does not necessarily have to occur. That is, the prime stimulus could activate visual representations of related words acting as a predictive signal for the visual system (see [27]).

Another explanation is based on the data-analysis method used in many studies. That is, most studies only perform a standard repeated measures ANOVA on (log-transformed) suppression times averaged across stimuli (i.e., the so-called F_1_ test). In psycholinguistics, this has been referred to as the “language-as-fixed-effect” fallacy [52] and incorporating stimulus as a random effect is standard in psycholinguistics nowadays. The importance of this practice has recently been demonstrated by Barr et al. [53] in a simulation study. In short, they showed that performing only an F_1_ test dramatically increases Type 1 errors especially for between-item manipulations (in that respect, it is interesting to remark that a classical repeated measures F_1_ analysis on the data of the main experiment of Experiment 2 yielded a marginally significant effect of word type (*F*_1_(1,19) = 3.3, *p* = .09)). Furthermore, in order to quantify the evidence in favor of one or the other model, statistical inference in this study was done in a Bayesian framework, which has shown to be more conservative than traditional null hypothesis significance testing with respect to the strength of the evidence for an effect [54]. This allows one to quantify evidence in favor of the hypothesis that no semantic processing occurs under CFS, while traditional test cannot confirm the null hypothesis [22], [55].

## Conclusion

In this study, the extent to which words are semantically processed in the absence of awareness (induced by CFS) was studied. In Experiment 1, no evidence was obtained for differential processing between word and pseudo-word stimuli nor a modulation of suppression time of words by word frequency. In Experiment 2, the absence of these effects was replicated. In contrast, a control experiment with a simpler stimulus showed that large white discs break suppression faster than small white discs. Finally, Experiment 3 replicated the face inversion effect, thus ruling out the possibility that the null effects were merely caused by our experimental set-up being insensitive to any high-level manipulation. These results were explained from the perspective that the suppressed stimuli might not have been processed up to the level at which semantic information is usually extracted. Alternatively, due to the instability of suppression times within and between participants, b-CFS might be an ill-suited paradigm to study unconscious semantic processing of words.

## Supporting Information

Figure S1 **Results of Experiment 1.** (A) The bar plot indicates mean suppression times for words and pseudo-words. The dots show the mean suppression time for each participant (connected dots refer to the same participant). (B) Scatter plot depicting the (absence of a) relationship between word frequency and suppression time. The data points refer to mean suppression time for each item averaged across participants. The black line refers to the posterior estimate of the relationship between word frequency and suppression time based on a mixed-effects model fit (with the BayesFactor package) with participants and words as crossed random effects and word frequency as a fixed effect.
(TIFF)

Figure S2 **Results of Experiment 2.** (A) The bar plot depicts the mean suppression times for each condition. The dots refer to mean suppression times per participant (connected dots refer to the same participant). (B) Mean suppression times for the control experiment. The bar plot depicts the grand mean for both conditions whereas the dots refer to single participants (connected dots refer to the same participant). (C) Scatter plot depicting the (absence of a) relationship between word frequency and suppression times for upright (black) and inverted (gray) words. The black and gray lines (hardly discernible) refer to the estimates of the relationship between word frequency and suppression time after a mixed-effects model fit with subject and word as crossed random effects and word frequency and inversion as fixed effects.
(TIFF)

Figure S3 **Results of Experiment 3.** The bar plot indicates mean suppression times for upright and inverted faces. The dots show the mean suppression time for each participant (connected dots refer to the same participant).
(TIFF)

Table S1 **Point Estimates, t-values and 95% Confidence Intervals of the Fixed Effects in Experiment 1 and 2.** *Note*. See Table 2 for an explanation of the models. One can consider the effect of a variable significant (i.e., *p*<.05, two-tailed) if the absolute value of the t-statistic is above 1.96. However, as Barr et al. [47] showed, this approach is very error-prone in the context of a frequentist hypothesis test using only random intercepts.
(DOCX)
