# Supplementary figures and images for: Frequent Words Do Not Break Continuous Flash Suppression Differently from Infrequent or Nonexistent Words: Implications for Semantic Processing of Words in the Absence of Awareness
Source: PLoS One. 2014 Aug 12;9(8):e104719. doi: 10.1371/journal.pone.0104719 (PMC4130538; doi:10.1371/journal.pone.0104719)

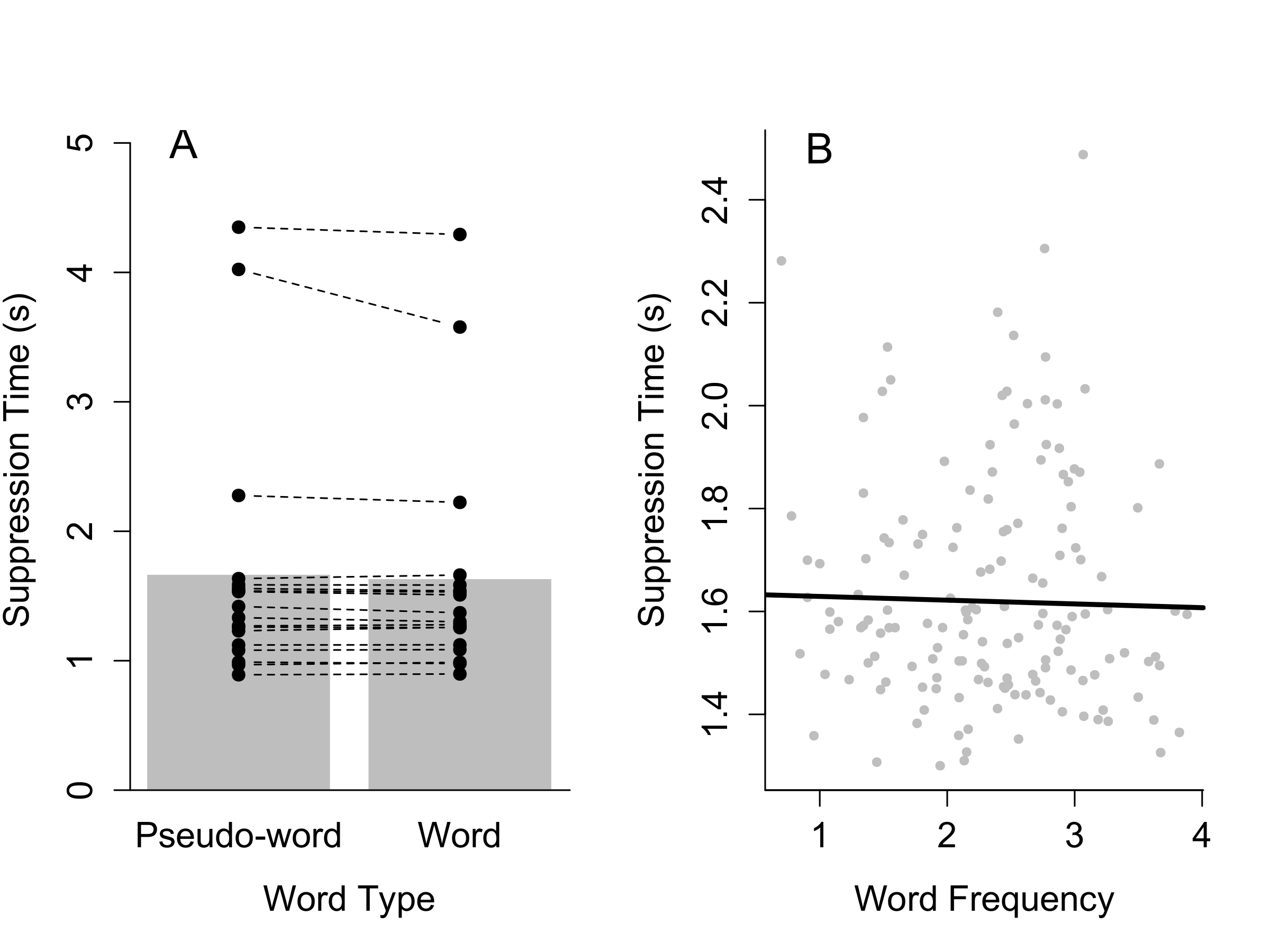

Supplement: Figure S1 — Results of Experiment 1. (A) The bar plot indicates mean suppression times for words and pseudo-words. The dots show the mean suppression time for each participant (connected dots refer to the same participant). (B) Scatter plot depicting the (absence of a) relationship between word frequency and suppression time. The data points refer to mean suppression time for each item averaged across participants. The black line refers to the posterior estimate of the relationship between word frequency and suppression time based on a mixed-effects model fit (with the BayesFactor package) with participants and words as crossed random effects and word frequency as a fixed effect. (TIFF) [file pone.0104719.s001.tiff]

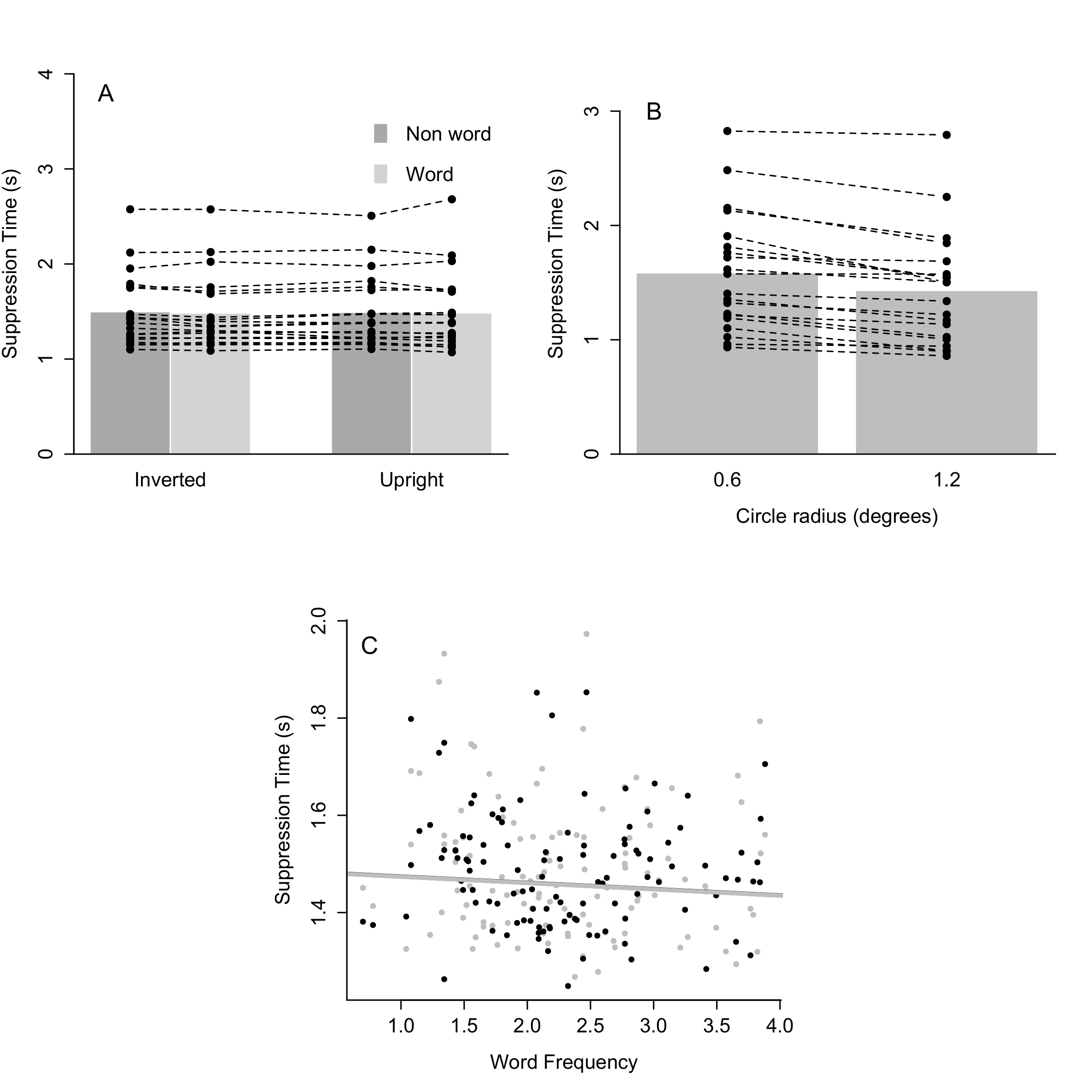

Supplement: Figure S2 — Results of Experiment 2. (A) The bar plot depicts the mean suppression times for each condition. The dots refer to mean suppression times per participant (connected dots refer to the same participant). (B) Mean suppression times for the control experiment. The bar plot depicts the grand mean for both conditions whereas the dots refer to single participants (connected dots refer to the same participant). (C) Scatter plot depicting the (absence of a) relationship between word frequency and suppression times for upright (black) and inverted (gray) words. The black and gray lines (hardly discernible) refer to the estimates of the relationship between word frequency and suppression time after a mixed-effects model fit with subject and word as crossed random effects and word frequency and inversion as fixed effects. (TIFF) [file pone.0104719.s002.tiff]

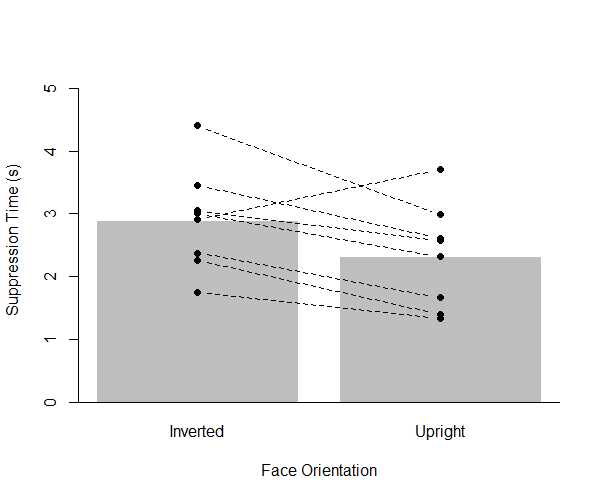

Supplement: Figure S3 — Results of Experiment 3. The bar plot indicates mean suppression times for upright and inverted faces. The dots show the mean suppression time for each participant (connected dots refer to the same participant). (TIFF) [file pone.0104719.s003.tiff]
