# Supplementary material for: Frequent Words Do Not Break Continuous Flash Suppression Differently from Infrequent or Nonexistent Words: Implications for Semantic Processing of Words in the Absence of Awareness
Source: PLoS One. 2014 Aug 12;9(8):e104719. doi: 10.1371/journal.pone.0104719 (PMC4130538; doi:10.1371/journal.pone.0104719)
Supplement: Table S1 — Point Estimates, t-values and 95% Confidence Intervals of the Fixed Effects in Experiment 1 and 2. Note. See Table 2 for an explanation of the models. One can consider the effect of a variable significant (i.e., p<.05, two-tailed) if the absolute value of the t-statistic is above 1.96. However, as Barr et al. [47] showed, this approach is very error-prone in the context of a frequentist hypothesis test using only random intercepts. (DOCX) [file pone.0104719.s004.docx]

| Model | Experiment 1 | | |  | | Experiment 2 | | | |
| --- | --- | --- | --- | --- | --- | --- | --- | --- | --- |
|  | Estimate | t | 95% CI | |  | | Estimate | t | 95% CI |
| (1) |  |  |  | |  | |  |  |  |
| *Mu* | 0.353 | 3.818 | [0.167 ; 0.538] | |  | | 0.331 | 6.651 | [0.231 ; 0.431] |
| *Pseudo/Non - word* | 0.003 | 0.696 | [-0.005 ; 0.011] | |  | | 0.003 | 1.653 | [-0.0006 ; 0.007] |
| *Word* | -0.003 | -0.696 | [-0.011 ; 0.005] | |  | | -0.003 | -1.653 | [-0.007 ; 0.0006] |
| *Inverted* | NA | NA | NA | |  | | 0.002 | 0.765 | [-0.002 ; 0.006] |
| *Upright* | NA | NA | NA | |  | | -0.002 | -0.765 | [-0.006 ; 0.002] |
|  |  |  |  | |  | |  |  |  |
| (2) |  |  |  | |  | |  |  |  |
| *Mu* | 0.350 | 3.841 | [0.167 ; 0.541] | |  | | 0.328 | 6.495 | [0.227 ; 0.429] |
| *Word Frequency* | -0.007 | -1.209 | [-0.019 ; 0.005] | |  | | -0.007 | -1.227 | [-0.019 ; 0.004] |
| *Inverted* | NA | NA | NA | |  | | 0.001 | 0.499 | [-0.004 ; 0.007] |
| *Upright* | NA | NA | NA | |  | | -0.001 | -0.499 | [-0.007 ; 0.004] |
|  |  |  |  | |  | |  |  |  |
| (3) |  |  |  | |  | |  |  |  |
| *Mu* | 0.352 | 3.847 | [0.168 ; 0.536] | |  | | 0.329 | 6.542 | [0.228 ; 0.430] |
| *Word Frequency* | -0.012 | -1.416 | [-0.028 ; 0.004] | |  | | -0.003 | -0.448 | [-0.017 ; 0.011] |
| *Inverted* | NA | NA | NA | |  | | 0.001 | 0.518 | [-0.004 ; 0.007] |
| *Upright* | NA | NA | NA | |  | | -0.001 | -0.518 | [-0.007 ; 0.004] |
| *Pixel Density* | -0.016 | -2.728 | [-0.028 ; -0.005] | |  | | -0.037 | -7.045 | [-0.047 ; -0.027] |
| *Trial* | -0.053 | -9.714 | [-0.064 ; -0.042] | |  | | -0.051 | -18.120 | [-0.057 ; -0.046] |
| *Age of Acquisition* | -0.008 | -1.001 | [-0.025 ; 0.008] | |  | | -0.004 | -0.577 | [-0.017 ; 0.009] |
| *Concreteness* | -0.008 | -1.062 | [-0.022 ; 0.006] | |  | | -0.005 | -0.876 | [-0.017 ; 0.006] |
|  |  |  |  | |  | |  |  |  |
| (4) |  |  |  | |  | |  |  |  |
| *Mu* | 0.354 | 3.822 | [0.168 ; 0.540] | |  | | 0.332 | 6.674 | [0.232 ; 0.431] |
| *Pixel Density* | -0.018 | -4.142 | [-0.027 ; -0.010] | |  | | -0.036 | -7.373 | [-0.045 ; -0.026] |
| *Trial* | -0.060 | -15.079 | [-0.067 ; -0.052] | |  | | -0.052 | -25.996 | [-0.056 ; -0.048] |

Table S1

*Point Estimates, t-values and 95% Confidence Intervals of the Fixed Effects in Experiment 1 and 2.*

*Note*. See Table 2 for an explanation of the models. One can consider the effect of a variable significant (i.e., *p* < .05, two-tailed) if the absolute value of the t-statistic is above 1.96. However, as Barr et al. [47] showed, this approach is very error-prone in the context of a frequentist hypothesis test using only random intercepts.
